# Stabilizing synchrony by inhomogeneity

**DOI:** 10.1038/srep13854

**Published:** 2015-09-04

**Authors:** Ehsan Bolhasani, Alireza Valizadeh

**Affiliations:** 1 Institute for Advanced Studies in Basic Sciences, Department of physics, Zanjan, 45137-66731, Iran; 2 Institute for Research in Fundamental Sciences, School of Cognitive Sciences, Niavaran, Tehran, 19857, Iran

## Abstract

We show that for two weakly coupled identical neuronal oscillators with strictly positive phase resetting curve, isochronous synchrony can only be seen in the absence of noise and an arbitrarily weak noise can destroy entrainment and generate intermittent phase slips. Small inhomogeneity–mismatch in the intrinsic firing rate of the neurons–can stabilize the phase locking and lead to more precise relative spike timing of the two neurons. The results can explain how for a class of neuronal models, including leaky integrate-fire model, inhomogeneity can increase correlation of spike trains when the neurons are synaptically connected.

Synchronization is observed frequently in the vast variety of physical, chemical, industrial, and biological complex systems, from coupled pendulum clocks to neuronal populations in the nervous system^1^,^2^,^3^,^4^,^5^,^6^. In these systems, ability to exhibit synchronous or phase locked oscillations is the foundation of the emergent behaviors which are the basis for the functionality of the system. While the existence of robust synchronization is important in real systems with different sources of noise and uncertainties in parameters, robustness and stability of this behavior is of a central importance.

Recordings of multi-neuron spike trains have revealed significant interdependencies between the firing of different neurons in a population^7^,^8^,^9^,^10^,^11^,^12^. Synchronous oscillations are found in many brain regions and excessive synchrony is a hallmark of neurological disorders such as epilepsy and Parkinson’s disease^13^. The functional role of the correlation in neural coding has been debated in recent years^14^,^15^,^16^,^17^. Synchrony itself may encode information directly^6^,^10^,^12^,^18^,^19^ and may underly feature binding^20^. Synchronous firing of the neurons in one region serves to reliably transmit signals to upstream regions^21^,^22^,^23^ and synchrony between different regions can prepare dynamic channels for communication^24^,^25^,^26^. Beyond their functional role, it is also important to understand how correlation and synchrony depend on the biophysical parameters of the neurons and the network.

In a population of neurons, correlation between the spike trains of any two neurons can arise from the shared input they receive^27^,^28^,^29^, or from the presence of direct synaptic connections between neurons^30^,^31^,^32^. In both cases the collective state of the system depends on the parameters of the neurons, e.g., the type of excitability of the neurons^33^, and the parameters of the connections such as delay^34^. Physiological heterogeneity can destabilize both coupling-induced and correlation-induced synchronization^35^,^36^,^37^. In the classical models of synchronization, collective state of a system of coupled oscillators is determined by the outcome of rivalry between synchronizing effect of connections and desynchronizing effect of inhomogeneity^2^, but there are examples of the systems in which synchrony is enhanced by inhomogeneity^38^,^39^,^40^,^41^. Recently we have shown that small inhomogeneity can increase correlation between spike trains of two coupled neurons^42^. In this study we develope a framework for the correlation of weakly coupled phase oscillators with a given phase sensitivity when they are driven by small amplitude noises. We show that for identical pulse coupled type-I oscillators, synchronized state can be seen in the absence of noise, but it is destroyed by an arbitrarily weak noise. Small inhomogeneity can stabilize phase locking by providing an asymmetric basin of attraction around the stable phase-locked state. Increasing inhomogeneity the effective basin of attraction of the locked state increases and the systems shows more robust locking. This results in a sharper probability distribution function (PDF) for the time difference between spiking of two neurons in the presence of weak noise. We have also shown that while for the model neurons with biologically realistic phase response curve (PRC), the time difference between the spikes of two neurons in the stable state increases with inhomogeneity, in the case of the leaky integrate-fire (LIF) neurons, they lock in almost zero phase lag for sufficiently small values of inhomogeneity. By solving Fokker-Planck equation we also find the most probable phase difference between spike times of the two neurons and will show that it does not coincide with the stable point of the deterministic equations. Although the synchrony in the general can be assigned both to the inphase firing and to the firing of the neurons with a non-zero phase lag, in the following we call the latter case by “phase-locked” state and use the term “synchronized” for inphase firing of the neurons.

## Methods

Our model comprises two bidirectionally coupled neuronal oscillators receiving suprathreshold constant currents (*I*_1_ and *I*_2_ with mismatch Δ*I*) as well as small amplitude independent stochastic inputs. The evolution of the state vector of the oscillators *X*_*i*_, *i* = 1,2 can be described by
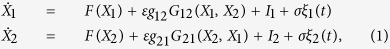
where *F* governs the internal dynamics of the neurons, *G* determines the synaptic connections, *ξ* is Gaussian white noise with zero mean and unit variance, and *ε* and *σ* are small values which scale strength of the couplings and the stochastic inputs, respectively. We assume that the unperturbed systems 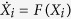 have asymptotically stable limit cycles, 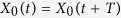, so that a phase variable can be defined in the neighborhood of the limit cycle. In the regime of weak coupling and weak noise we can apply the standard phase reduction^2^,^43^ to the Langevin equations above. The system can be described by a set of Itô stochastic differential equations (see supplementary material):
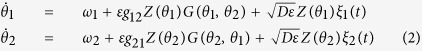


where *Z*(*θ*) is the infinitesimal phase-response curve (PRC)^44^. We assume that the natural frequencies have a small difference *ω*_1_ − *ω*_2_ = Δ*ω* and the noise and the coupling influence only the first (voltage) variable of the state vector of the neural oscillators. In our model the neurons communicate via pulsatile signals 
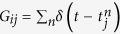
, where *δ* is Dirac’s delta function and 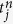 is the instant of *n*^*th*^ firing of the neuron *j*. These pulses idealize the communication signals which are short compared to the intrinsic time scale of the oscillators and are used to model diverse systems such as populations of flashing fireflies, plate tectonics in earthquakes, and the networks of spiking neurons in the brain^45^,^46^,^47^,^48^,^49^. It is assumed that the coupling and noise terms are of the same order, sufficiently weak such that the phase representation for the intrinsic dynamics of isolated oscillators remains valid for the coupled noise-driven oscillators. Using the method of averaging^50^ we derive the equation of motion for the phase difference *ϕ* = *θ*_1_ − *θ*_2_ (see supplementary material):
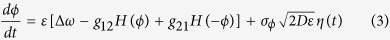
where 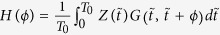 and the coefficient 
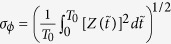
 comes from averaging the noisy phase equations^2^. Here 
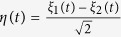
 is itself a Gaussian white noise with zero mean and unit variance.

## Results

For two coupled neuronal oscillators, described by Eq. (1) we study the distribution of the phase differences in the regime of weak coupling and weak noise. In this regime dynamics of each neuron can be described by single phase variable and the phase difference of the neurons obeys Langevin equation (3). We restrict the study to type-I oscillators and first we discuss on the deterministic version of Eq. (3) with *D* = 0. If *H* is an even function of *ϕ*, e.g. for the quadratic integrate-fire (QIF) oscillators^51^, the effective coupling term would be Δ*gH*(*ϕ*) with Δ*g* = *g*_21_ − *g*_12_. In this case the effective coupling strength is determined by the difference of the two connection strengths and the symmetric connection has no effect on the relative dynamics of the oscillators. For the oscillators with an *oblique* PRC, e.g. the LIF oscillators, the coupling term can be non-zero for symmetric connections (see supplementary material Fig. S1).

**Figure 1:**
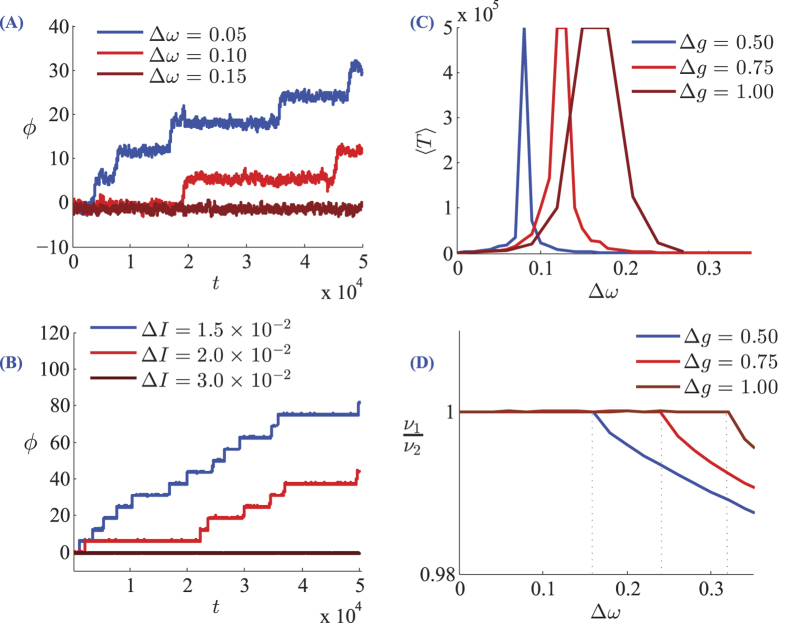
(**A,B**) Representative examples of the evolution of the phase difference of two neurons for three different values of mismatch in intrinsic frequencies. Larger values of mismatch have led to fewer phase slips. In (**A**) neurons are phase oscillators with canonical type-I phase sensitivity and in (**B**) the results are presented for LIF neurons. (**C**) The mean escape time is plotted against frequency mismatch. Increasing effective coupling constant Δ*g* = *g*_1_ − *g*_2_ the maximum escape time is seen in larger values of frequency mismatch. In (**D**) the ratio of the firing rates of the coupled neurons is plotted. For large values of mismatch the fixed point of 1:1 locking vanishes.

The fixed point of Eq. (3) with *D* = 0 is the solution of Δ*ω* = *g*_12_*H*(*ϕ*) − *g*_21_*H*(−*ϕ*). For QIF oscillators *Z*(*ϕ*) = 1 − *cos*(*ϕ*) with asymmetric connections Δ*g* ≠ 0, when the oscillators are identical Δ*ω* = 0, the zero-lag synchrony *ϕ* ≠ 0 is a fixed point, but the system is in the point of a saddle-node bifurcation. Such fixed points are unstable, but in 
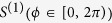
, when no other fixed points are present in the phase space, they attract all the points in the phase space in infinite time^52^. Consequently, in the absence of noise the oscillators can synchronize isochronously but a weak noise can destroy synchrony and lead to phase slips. Mismatch in the intrinsic firing rates of the neurons, stabilizes the fixed point through a saddle-node bifurcation while moves the fixed point away from zero. For small mismatch, this provides an asymmetric basin of attraction which is vulnerable to sufficiently large perturbations in one direction around the fixed point. In the presence of noise the system shows epochs of intermittent locking between which the relative phase of the oscillators slips by one cycle, while the mean escape time from locked state increases with frequency mismatch (see Fig. 1A,B). This increase in the escape time, is related to expansion of the effective basin of attraction of the stable fixed point of the system. The maximum mean escape time is seen in a certain value of mismatch (Fig. 1C) and further increase of the mismatch shrinks the basin of the attraction of the fixed point in the opposite side which decreases the escape time. For larger mismatch 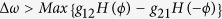, the fixed point corresponding to 1:1 locked state will disappear through another saddle-node bifurcation (see supplementary material Fig. S1). Two points are worthy of note about generality of the above arguments: First, for the biological neuronal models, PRC is zero near the spike time of the neuron and for type-I neurons with a well-behaved non-negative PRC, this means that the slope of the PRC has opposite signs in the two sides of the spike time which is usually is taken as phase zero. Therefore, for a coupled pair of such neurons (when the neurons are identical), the synchronized state is a fixed point which is attracting but is not stable and the above arguments hold for all the neuronal models which have this property, e.g., Wang-Bazsaki (WB) neurons^53^. Second, for the type-II oscillators the PRC has negative and positive parts and this means that a well-behaved PRC has at least one zero-crossing point with negative slope and there exists a stable phase-locked fixed point for coupled identical type-II oscillators. For such systems small inhomogeneity does not change the stability of the fixed point but it can expand or shrink its basin of attraction.

To give more concrete results on the impact of the inhomogeneity on the correlation of the spike trains of the neuronal oscillators in presence of noise, we derive the Fokker-Planck equation for the distribution of the phase differences of two neurons, described by Eq. (3). We rewrite Eq. (3) in a more closed form
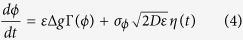
where 
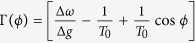
. The corresponding Fokker-Planck equation takes the form:
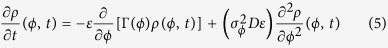
where *ρ*(*ϕ*,*t*) is the distribution of the phase differences. Stationary solution of this equation with periodic boundary condition is:
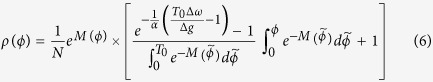
where 
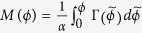
. Also, *N* is the normalization factor so that 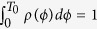 and 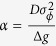 is the ratio of noise intensity to the coupling strength^54^.

**Figure 2:**
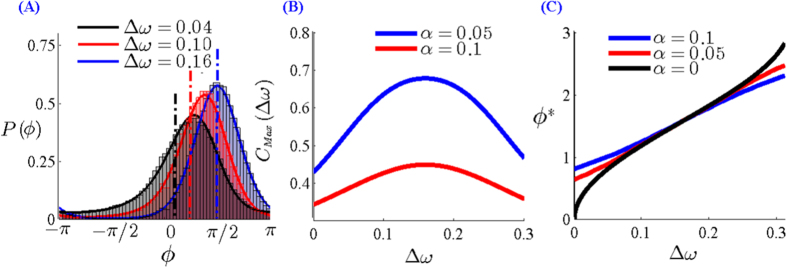
(**A**) The steady state phase difference distributions *ρ*(*ϕ*) for three levels of heterogeneity. Distributions have become narrower as mismatch is increased. Solid lines show the analytic result Eq. (6) and the bar graph presents the numerical results by direct integration of Eq. (2). Dashed vertical lines show the position of the fixed points of deterministic equations. (**B**) The maximum value of *ρ*(*ϕ*) is plotted against frequency mismatch for two different values of the ratio of noise strength to effective coupling 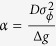. (**C**) The most probable phase difference (shown by dashed line in A) is shown for three values of α. In the presence of noise (α ≠ 0) the most probable phase difference is different from the fixed point of the deterministic equations (black curve α = 0).

Figure 2A shows the steady state phase difference distribution for different values of the frequency mismatch for QIF neuronal oscillators. It can be seen that the distribution becomes narrower (with a more pronounced peak) with increasing frequency mismatch while the neurons remain in 1:1 locked state, i.e. for the mismatch in the range 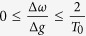. This reflects a larger basin of attraction for the locked state when mismatch is increased from zero. Most robust locking occurs for 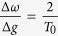 when the basin of attraction is symmetric around the stable fixed point. Furthermore, the asymmetry of the basin of attraction causes the distribution of the phase differences not to peak in the fixed point of the deterministic equation, determined by 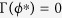. In turn, in presence of noise the location of maximum phase difference satisfies,
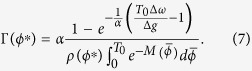
which is derived by taking the derivative of *ρ*(*ϕ*) with respect to *ϕ*, equal to zero. The location of the most probable phase difference as a function of mismatch, determined by Eq. (7), is plotted in Fig. 2C for different values of the noise amplitude as well as for the noiseless system which coincides with the location of the fixed point. Presence of noise inclines the distribution to larger phase differences for small values of frequency mismatch. The maximum difference between the location of most probable phase difference between noiseless state and noisy state is seen near 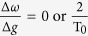 which reflects the most asymmetric basin of attraction for the locked state and in turn the locations coincide when ϕ^*^ = *π*/2 where the basin of attraction is symmetric.

**Figure 3:**
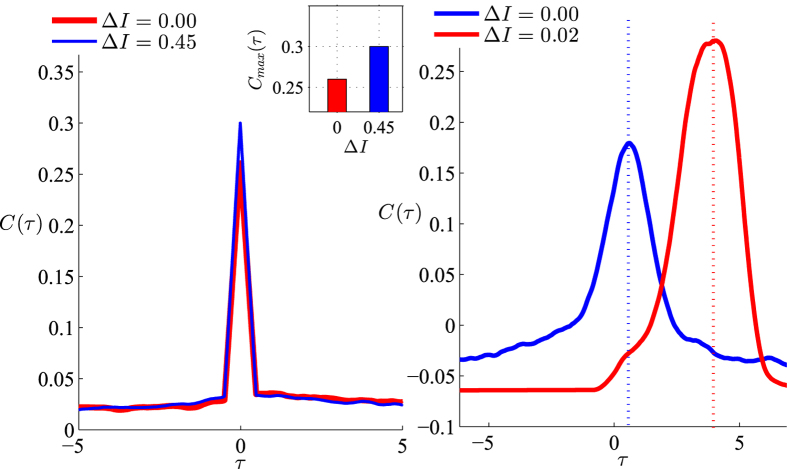
The cross-correlogram of spike trains of two neurons *C*(*τ*) shows that in presence of the firing rate mismatch cross correlation is increased. The left panel shows the results for LIF neurons and the level of maximum correlation is shown in the inset to highlight the increase due to the inhomogeneity. In the right panel the results are presented for two Wang-Buzsaki (WB) neurons. The parameters for both simulations are given in the supplemantary material.

Pfeuty *et al.*^55^ have introduced a variable *S*_*i*_(*t*) which is equal to 1/*δ* when a neuron has fired a spike in a time bin of size *δ* about time t and is equal to 0 otherwise^55^. For sufficiently small *δ* the time average of *S*_*i*_ is the average firing rate of neuron *i*. It is shown that the normalized cross-correlogram (CC) of this variable which is the probability of the firing of the neuron 2 in a time bin of size *δ* and the delay *τ* after a spike of neuron 1, is related to the phase difference probability distribution function *ρ*(*ϕ*) through
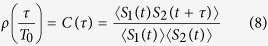
where 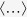 indicates averaging over time. A peak in CC at a time lag *τ* shows phase locking of the activity of the neurons. The sharper CC is indicator of a tighter locking. To illustrate this effect we provide an expression for the maximum value of the distribution function (or CC) as a function of frequency mismatch:
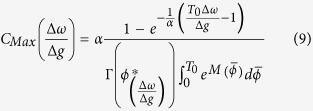


The above equation is the same as Eq. (7) by substituting *ρ*(ϕ^*^) with 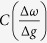. Figure 2B shows maximum value of cross-correlation versus frequency mismatch for two canonical type-I phase oscillators for different values of noise to coupling ratio which is resulted from direct integration of Eq. (7). The result shows that the maximum cross-correlation of the spike trains of the oscillators would be also maximum when the neurons are not identical. This is a consequence of more precise relative spike timing of the two neurons in presence of inhomogeneity. In Fig. 3 we have shown the cross-correlogram for LIF oscillators and also for the WB model neurons. While for both the models small inhomogeneity increases correlation of the spike trains, for the WB model neurons inhomogeneity also moves the maximum correlation to the non-zero phase lags similar to the canonical type-I phase oscillators.

## Discussion

While disorder usually acts against synchrony in the networks of coupled autonomous oscillators, there are intriguing examples in which a source of disorder enhances order in the behavior of a dynamical system by enhancing the response of the system to the external signal as a resonance-like behavior^38^,^39^,^40^,^56^. As an example of the order induced by inhomogeneity, in this study we have shown that in a minimum system of two synaptically connected neuronal oscillators, more precise relative spike timing can be achieved when the neurons receive different levels of inputs and have different intrinsic firing rates. It is shown that inhomogeneity can increase the effective basin of attraction of the fixed point which determines the phase locked state and hence makes the locking more robust against noise. Consequently, cross-correlation of spike trains of the noise driven neurons increases in presence of mismatch in intrinsic firing rates of neurons.

While the results are presented for neuronal oscillators, they can find application in general context of coupled limit cycle oscillators. Our analytic results showed that for a class of coupled limit cycle oscillators with non-nagetive phase response curves, the locked state is more robust against noise when the osillators are not indentical, provided that the PRC is zero for certain values of the phase of the oscillators. For type-II oscillators the PRC takes both negative and postive values and the phase-locked state would be stable for two coupled identical oscillators. In this case small inhomogeneity does not change the stability of the fixed point corresponding to the locked state, although it can still increase the correlation of the spike trains of the neurons by expanding the basin of attraction of the fixed-point.

It has been shown that the complete synchonization is possible for coupled chaotic oscillators when they are identical and coupled by instantaneous connections^57^,^58^. Presence of inhomogeneity and delayed connections lead to other types of synchrony such as phase or lag synchronization^58^,^59^. To the best of our knowledge, the notion of PRCs is not extended to the chaotic oscillators but our results may be applicable to the certain types of the chaotic oscillators which endergo phase advance due to the external excitations, like type-I limit cycle oscillators. Since the dynamics of the chaotic oscillators is similar to the noisy limit cycle oscillators, we expect the possibility of the enhacement of the synchrony by inhomogeneity for chaotic oscillators, even in the absence of any external noise.

## Additional Information

**How to cite this article**: Bolhasani, E. and Valizadeh, A. Stabilizing synchrony by inhomogeneity. *Sci. Rep.* **5**, 13854; doi: 10.1038/srep13854 (2015).

## Supplementary Material

Supplementary Information
