# Supplementary material for: Stabilizing synchrony by inhomogeneity
Source: Sci Rep. 2015 Sep 4;5:13854. doi: 10.1038/srep13854 (PMC4559804; doi:10.1038/srep13854)
Supplement: Supplementary Information [file srep13854-s1.pdf]

Supplementary Material for "Stabilizing synchrony by inhomogeneity"

Ehsan Bolhasani[1,2] and Alireza Valizadeh[1,2]

[1]Department of physics, Institute for Advanced Studies in Basic Sciences, Zanjan, Iran.
[2]School of Cognitive Sciences, Institute for Studies in Theoretical Physics and Mathematics, Niavaran, Tehran, Iran.

(Dated: July 20, 2015)

PHASE MODEL FOR WEAKLY COUPLED NOISY OSCILLATORS

Our model comprises two bidirectional coupled neurons receiving suprathreshold constant currents as well as uncorrelated stochastic inputs. The general form of equations describing this model is given by

$$\dot{X}_1 = F(X_1) + I_1 + \epsilon g_{12} G_{12}(X_1, X_2) + \sigma\xi_1(t)$$
$$\dot{X}_2 = F(X_2) + I_2 + \epsilon g_{21} G_{21}(X_2, X_1) + \sigma\xi_2(t) \tag{S1}$$

where X_i is a N-dimensional state vector containing the membrane potential and gating variables. For example in the Hodgkin-Huxley (HH) model [S1], $X = [V, m, h, n]^T$ and in the Leaky Integrate-and-Fire (LIF) model [S2], $X = V$. $F(X)$ defines the internal dynamics of the neuron i. $G_{ij}(X_i, X_j)$ determines functional form of synaptic connection from neuron j to neuron i. For example in the case of pulse coupled synapses, as we used in this letter it would be

$$G_{ij}\Big(X_i(t), X_j(t)\Big) = \sum_n \delta\left(t - t_j^n\right) \tag{S2}$$

where t_j^n is the instant of nth spike of neuron j and $\delta(t)$ is the Dirac's delta function. g_{ij} shows the synaptic weight from neuron j to neuron i which is scaled by a small factor ε so that the weakly coupled oscillators approximation is valid.

Each neuron receives suprathreshold constant current I_i with mismatch $\Delta I = I_1 - I_2$ as well as an independent Gaussian white noise characterized by its mean and auto-correlation, and its cross-correlation with the other neuron's input

$$\langle \xi_i(t) \rangle = 0, \tag{S3}$$

$$\langle \xi_i(t)\, \xi_j(t') \rangle = \delta_{ij}\delta(t - t'). \tag{S4}$$

We assume that at the absence of synaptic connections and noise, dynamics of each isolated neuron with the input current I_i given by

$$\dot{X}_i = \bar{F}(X_i) = F(X_i) + I_i \tag{S5}$$

has a T-periodic limit cycle solution $X_0(t)$. Every point on the limit cycle X_0 can be characterized by a single phase variable θ. On the limit cycle $X = X_0(\theta)$, which its inverse maps each point on the limit cycle to a unique phase, $\theta = X_0^{-1}(X) = \Phi(X)$. The phase of each oscillator along the limit cycle is then

$$\theta_i(t) = (\omega_i t + \phi_i) \quad mod \quad 2\pi \tag{S6}$$

where the relative phase ϕ_i is a constant which is determined by the initial conditions [S3]. From the definition of phase (Eq. S6), the rate of the phase change on the limit cycle is

$$\frac{d\theta}{dt} = \omega. \tag{S7}$$

So by means of the chain role we obtain the following useful relation:

$$\frac{d\theta}{dt} = \nabla_X \Phi(X_0(t)) . \frac{dX_0}{dt} = \nabla_X \Phi(X_0(t)) . \bar{F}(X) = \omega \tag{S8}$$

Where $\nabla_X \Phi$ is the gradient of Φ with respect to the vector state variable X.

Using the notion of asymptotic phase and isochrons (See Refs. [S4] and [S3]) the phase of oscillation $\theta = \Phi(X)$ can also be defined for all the points X in the neighborhood of the limit cycle. By this concept we can introduce $Z(\theta) = \nabla_X \Phi$ as the infinitesimal phase response curve (iPRC): If the neuron on the limit cycle which is in the state $X(t)$, receives a small vector perturbation εU in a short time interval Δt, immediately after the perturbation the state of the neuron jumps to $X(t) + \varepsilon U$. So the resulted phase change is

$$\Delta\theta \simeq \nabla_X \Phi \left(X(\theta)\right) . \varepsilon U \tag{S9}$$

Therefore in the presence of perturbation Eq. (S8) changes as

$$\frac{d\theta}{dt} \simeq \omega + Z(\theta).\varepsilon U. \tag{S10}$$

We consider the coupling and noise terms in Eq. S1 as small perturbations on the limit cycle $\dot{X}_i = \bar{F}(X_i)$. We also assume that inherent frequency of neurons have a small difference of the order $\mathcal{O}(\varepsilon)$. So if $\sigma = \sqrt{D\varepsilon}$ ($D \sim \mathcal{O}(1)$) we can reduce high dimensional Eqs. S1 to two scalar equations which determine the evolution of the phase [S5–S8]

$$\dot{\theta}_1 = \omega_1 + \varepsilon g_{12} Z\left(\theta_1\right) G\left(\theta_1, \theta_2\right) + \sqrt{D\varepsilon} Z\left(\theta_1\right) \xi_1\left(t\right) \tag{S11}$$
$$\dot{\theta}_2 = \omega_2 + \varepsilon g_{21} Z\left(\theta_2\right) G\left(\theta_2, \theta_1\right) + \sqrt{D\varepsilon} Z\left(\theta_2\right) \xi_2\left(t\right) \tag{S12}$$

where $Z\left(\theta\right)$ is the infinitesimal phase response curve [S9] and $\varepsilon\Delta\omega = \omega_2 - \omega_1$. Assuming $X_i\left(t\right) \simeq X_0\left(\theta_i\left(t\right)\right)$ and by the change of variable $\theta_i\left(t\right) = \omega t + \phi_i\left(t\right)$, the equations for the evolution of the relative phase of the oscillators read:

$$\dot{\phi}_1 = -\frac{\varepsilon\Delta\omega}{2} + \varepsilon g_{12} Z\left(\phi_1\right) G\left(\phi_1, \phi_2\right) + \sqrt{D\varepsilon} Z\left(\phi_1\right) \xi_1\left(t\right) \tag{S13}$$
$$\dot{\phi}_2 = +\frac{\varepsilon\Delta\omega}{2} + \varepsilon g_{21} Z\left(\phi_2\right) G\left(\phi_2, \phi_1\right) + \sqrt{D\varepsilon} Z\left(\phi_2\right) \xi_2\left(t\right). \tag{S14}$$

We exploit the fact that ε is small to further reduce Eqs. S14. With a system of the form

$$\dot{x} = \varepsilon f\left(x, t\right). \tag{S15}$$

Averaging theory [S8, S10, S11] states that in Eq. S15, $x\left(t\right)$ can be replaced by its average over a period $\bar{x}$ and

$$\dot{\bar{x}} = \varepsilon \frac{1}{T} \int_0^T f\left(\bar{x}, t\right) dt. \tag{S16}$$

By applying averaging method on the Eqs. S14 we have

$$\dot{\phi}_1 = -\varepsilon\frac{\Delta\omega}{2} + \varepsilon\frac{g12}{T} Z\left(\phi_2 - \phi_1\right) + \sigma_\phi \sqrt{D\varepsilon}\xi_1\left(t\right) \tag{S17}$$
$$\dot{\phi}_2 = +\varepsilon\frac{\Delta\omega}{2} + \varepsilon\frac{g21}{T} Z\left(\phi_1 - \phi_2\right) + \sigma_\phi \sqrt{D\varepsilon}\xi_2\left(t\right) \tag{S18}$$

where the term $\sigma_\phi = \left(\frac{1}{T}\int_0^T \left[Z\left(\tilde{t}\right)\right]^2 d\tilde{t}\right)^{1/2}$ originates from averaging the noisy phase equations [S12], and $Z\left(\phi_i - \phi_j\right)$ comes from

$$H\left(\phi_2 - \phi_1\right) = \frac{1}{T}\int_0^T Z\left(\theta_1\right) G\left(\theta_1, \theta_2\right) d\theta_1 \tag{S19}$$
$$= \frac{1}{T}\int_0^T Z\left(\theta_1\right) \delta\left(\theta_2\right) d\theta_1 \tag{S20}$$
$$= \frac{1}{T}\int_0^T Z\left(t + \phi_1\right) \delta\left(t + \phi_2\right) dt \tag{S21}$$
$$= \frac{1}{T}\int_0^T Z\left(\tilde{t}\right) \delta\left(\tilde{t} - \phi_1 + \phi_2\right) d\tilde{t} \tag{S22}$$
$$= \frac{Z\left(\phi_2 - \phi_1\right)}{T}. \tag{S23}$$

without loss of generality we assumed that the phase is normalized so that $0 \leq \theta < T$, i.e., $\omega = 1$. By defining $\phi = \phi_2 - \phi_1$, we derive the following equation for the phase difference

$$\dot{\phi} = \varepsilon\Big(\Delta\omega + g_{21}H(-\phi) - g_{12}H(\phi)\Big) + \sigma_\phi\sqrt{2D\varepsilon}\eta\,(t) \tag{S24}$$

where $\sqrt{2}\eta\,(t) = \xi_2 - \xi_1$ and $\eta(t)$ is a Gaussian white noise with zero mean and unit variance.

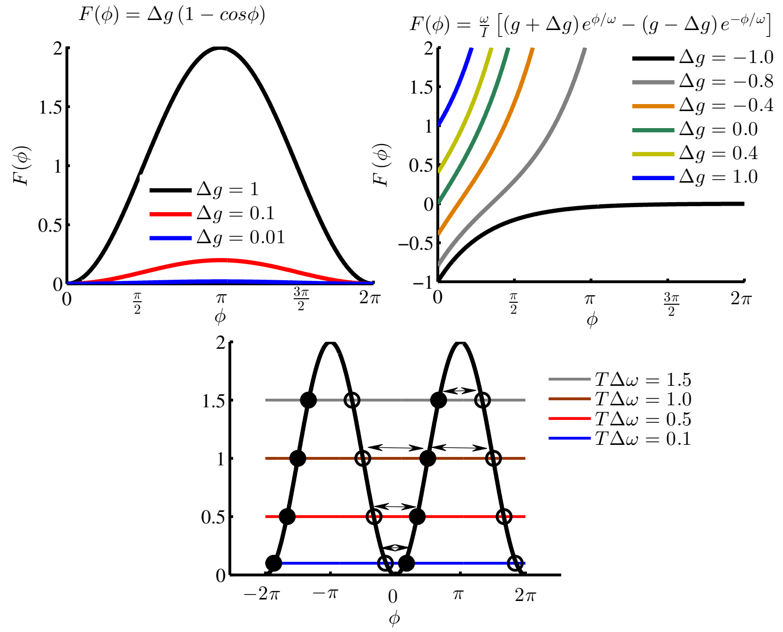

FIG. S1: Examples of $F\,(\phi) = g_{12}H\,(\phi) - g_{21}H\,(-\phi)$, for QIF oscillator (top-left) and LIF oscillator (top-right) for different values of difference of the synaptic strengths. For the LIF oscillators for which PRC is not an even function, the coupling term can be non-zero for symmetric connections whereas for QIF oscillators with an even PRC the effective coupling is determined by the difference of two reciprocal synaptic constants. In the lower panel we have shown how increasing the mismatch in the firing rates of the neurons stabilizes the fixed point and then expands its basin of attraction.

We have used three model neurons in our study: (i) Canonical type-I oscillators with $Z(\phi) = 1 - \cos(\phi)$, (ii) LIF oscillators which is described by $\dot{v} = I - v$ for $t \leq v_{Th}(= 1)$ and $lim_{\tau\to 0^+}v(t+\tau) = 0$ with

$$Z(\phi) = \frac{\omega}{I}exp\Big(\frac{\phi}{\omega}\Big), \quad 0 \leq \phi < 2\pi, \tag{S25}$$

where $\omega = 2\pi/[logI - log(I-1)]$ [S13], and (iii) the conductance based Wang-Buzsaki (WB) model as described at the end of the supplementary material [S14].

First we focus on the deterministic case of Eq. S24 with $D = 0$. For QIF oscillator, $Z\,(\phi) = 1 - \cos\,(\phi)$ is an even function of ϕ. Therefore it reduces to

$$\dot{\phi} = \varepsilon\left[\Delta\omega + \frac{\Delta g}{T}\Big(1 - \cos(\phi)\Big)\right] \tag{S26}$$

where $\Delta g = g_{21} - g_{12}$ is the effective coupling constant. In this case the most effective coupling is that which maximizes Δg. i.e., a unidirectional one and the symmetric connection leads to zero effective coupling $\Delta g = 0$. But for LIF neurons with uneven PRC Eq. S24 takes the form

$$\dot{\phi} = \varepsilon\left[\Delta\omega + \frac{\omega g_{21}}{IT}exp\Big(\frac{-\phi}{\omega}\Big) - \frac{\omega g_{12}}{IT}exp\Big(\frac{\phi}{\omega}\Big)\right] \quad 0 \leq \phi < 2\pi. \tag{S27}$$

Note that for the oscillators with an *oblique* PRC, e.g. the LIF oscillators, the effective coupling term can be non-zero for symmetric connections (see Fig. S1). The fix points of general equation S24 with $D = 0$ are the intersections of horizontal line $T\Delta\omega$ and the curve described by $F(\phi) = g_{12}Z(\phi) - g_{21}Z(-\phi)$.

For type-I phase oscillators we rewrite Langevin Eq. S24 as

$$\frac{d\phi}{dt} = \varepsilon Z g \Gamma(\phi) + \sigma_\phi \sqrt{2D\varepsilon}\eta(t) \tag{S28}$$

with $\Gamma(\phi) = \left[\frac{\Delta\omega}{\Delta g} - \frac{1}{T} + \frac{1}{T}\cos\phi\right]$. Corresponding Fokker-Planck equation [S15–S17] for the phase difference distribution $\rho(\phi, t)$ is

$$\frac{\partial \rho}{\partial t}(\phi, t) = -\varepsilon \frac{\partial}{\partial \phi}\left[\Gamma(\phi)\rho(\phi, t)\right] + \left(\sigma_\phi^2 D\varepsilon\right)\frac{\partial^2 \rho}{\partial \phi^2}(\phi, t). \tag{S29}$$

The stationary phase difference distribution satisfies

$$\frac{\partial \rho_0(\phi, t)}{\partial t} = 0, \tag{S30}$$

with the solution

$$\rho_0(\phi) = \frac{1}{N} e^{M(\phi)}\left[A\int_0^T e^{-M(\phi')}d\phi' + 1\right], \tag{S31}$$

where

$$M(\phi) = \frac{1}{\alpha}\int_0^\phi \Gamma\left(\bar{\phi}\right)d\bar{\phi}. \tag{S32}$$

N is a normalization factor so that $\int_0^T \rho(\phi)\,d\phi = 1$, and $\alpha = \frac{D\sigma_\phi^2}{\Delta g}$ is the ratio of noise intensity to the coupling strength. The constant A can be determined by the periodicity condition of ρ_0, that is, $\rho_0(0) = \rho_0(T)$. Therefore the final form of stationary solution is

$$\rho(\phi) = \frac{1}{N} e^{M(\phi)} \times \left[\frac{e^{-\frac{1}{\alpha}\left(\frac{T\Delta\omega}{\Delta g} - 1\right)} - 1}{\int_0^T e^{-M(\tilde{\phi})}d\tilde{\phi}}\int_0^\phi e^{-M(\tilde{\phi})}d\tilde{\phi} + 1\right]. \tag{S33}$$

In Figure 2A we have shown the result of analytic solution for steady-stat phase difference distributions S33 and that of direct numerical integration of of phase differential equations S12. For solving Eq. S33, we have used double int function of MATLAB. In simulation, we integrate Eqs. S12 with Euler method and save spike times of each neuron. Then we have used hist function in MATLAB to plot ρ.

It has been shown that [S18] in the weak coupling and weak noise limit, the cross-correlogram (CC) and the phase difference probability distribution function, $\rho(\phi)$, are related by

$$C(\tau) = \rho\left(\frac{\tau}{T}\right). \tag{S34}$$

The most probable phase difference of spiking of the neurons (location of the peak of the PDF in Fig. 2A) can be determined by differentiation of ρ with respect to ϕ. Derivative of ρ with respect to ϕ is

$$\frac{d\rho}{d\phi} = \frac{dM(\phi)}{d\phi} e^{M(\phi)}\left[\frac{e^{-\alpha^{-1}\left(\frac{T\Delta\omega}{\Delta g} - 1\right)} - 1}{\int_0^T e^{-M(\bar{\phi})}d\bar{\phi}}\int_0^\phi e^{-M(\bar{\phi})}d\bar{\phi} + 1\right] + e^{M(\phi)}\left[\frac{e^{-\alpha^{-1}\left(\frac{T\Delta\omega}{\Delta g} - 1\right)} - 1}{\int_0^T e^{-M(\bar{\phi})}d\bar{\phi}} e^{-M(\bar{\phi})}\right]. \tag{S35}$$

For $Z(\phi) = 1 - \cos(\phi)$, $M(\phi)$ would be

$$M(\phi) = \frac{1}{\alpha}\left(\frac{\Delta\omega}{\Delta g}\phi - \frac{1}{T}\phi + \frac{1}{T}\sin(\phi)\right), \tag{S36}$$

and Eq. S35 reduces to

$$\frac{d\rho}{d\phi} = \frac{1}{\alpha}\left(\frac{\Delta\omega}{\Delta g} - \frac{1}{T} + \frac{1}{T}\cos(\phi)\right) e^{M(\phi)}\left[\frac{e^{-\alpha^{-1}\left(\frac{T\Delta\omega}{\Delta g} - 1\right)} - 1}{\int_0^T e^{-M(\bar{\phi})}d\bar{\phi}}\int_0^\phi e^{-M(\bar{\phi})}d\bar{\phi} + 1\right] + \frac{e^{-\alpha^{-1}\left(\frac{T\Delta\omega}{\Delta g} - 1\right)} - 1}{\int_0^T e^{-M(\bar{\phi})}d\bar{\phi}}, \tag{S37}$$

therefore

$$0 = \frac{1}{\alpha}\left(\frac{\Delta\omega}{\Delta g} - \frac{1}{T} + \frac{1}{T}\cos(\phi^*)\right) e^{M(\phi^*)} \left[\frac{e^{-\alpha^{-1}\left(\frac{T\Delta\omega}{\Delta g}-1\right)} - 1}{\int_0^T e^{-M(\bar{\phi})} d\bar{\phi}} \int_0^{\phi^*} e^{-M(\bar{\phi})} d\bar{\phi} + 1\right] + \frac{e^{-\alpha^{-1}\left(\frac{T\Delta\omega}{\Delta g}-1\right)} - 1}{\int_0^T e^{-M(\bar{\phi})} d\bar{\phi}}, \quad \text{(S38)}$$

and

$$e^{M(\phi^*)} \left[\frac{e^{-\alpha^{-1}\left(\frac{T\Delta\omega}{\Delta g}-1\right)} - 1}{\int_0^T e^{-M(\bar{\phi})} d\bar{\phi}} \int_0^{\phi^*} e^{-M(\bar{\phi})} d\bar{\phi} + 1\right] = -\frac{e^{-\alpha^{-1}\left(\frac{T\Delta\omega}{\Delta g}-1\right)} - 1}{\frac{1}{\alpha}\left(\frac{\Delta\omega}{\Delta g} - \frac{1}{T} + \frac{1}{T}\cos(\phi^*)\right) \int_0^T e^{-M(\bar{\phi})} d\bar{\phi}}. \quad \text{(S39)}$$

By using equations S39 and S33 we have

$$\rho(\phi^*) = -\frac{1}{N} \frac{e^{-\alpha^{-1}\left(\frac{T\Delta\omega}{\Delta g}-1\right)} - 1}{\frac{1}{\alpha}\left(\frac{\Delta\omega}{\Delta g} - \frac{1}{T} + \frac{1}{T}\cos(\phi^*)\right) \int_0^T e^{-M(\bar{\phi})} d\bar{\phi}}, \quad \text{(S40)}$$

then ϕ^* is

$$\begin{aligned} \phi^*(\Delta\omega) &= \langle\phi\rangle \\ &= \int_0^T \phi \ \rho_{\Delta\omega}(\phi) d\phi. \end{aligned} \quad \text{(S41)}$$

We have used MATLAB function "int" to plot ϕ^* versus $\Delta\omega$ in Figure 2C using Eqs. S33, S41, and C_{max}; and ρ_{max} versus $\Delta\omega$ in Figure 2B by Eq. S40.

NEURONAL MODELS

In Figure 3 (left panel) we have plotted $C(\tau)$ for two leaky integrate-fire (LIF) neurons described by

$$\tau_m \dot{v_i} = v_{rest} - v_i + I_i + g_{ij} G(v_i, v_j) + \sqrt{D}\xi_i(t) \quad \text{(S42)}$$

with a reset condition $v_i(t^+ = V_r)$ if $v_i(t^- > V_{th})$. We integrated this equation for two pulse coupled neurons and calculated spike count for a time window of teh width $0.5mS$. Parameters are selected in accordance with experimental data [S19] as $\tau_m = 20\ ms$, $v_{res} = -74\ mV$, $V_{th} = -54\ mV$, $v_r = -60\ mV$, $I = 25.0 + \Delta I\ mV$, $g_{ij} = 1\ mV$ and $D = 1.0$.

In figure 3 (right panel) we have used the conductance-based Wang-Buzsaki (WB) model [S14] which is widely used for simulation of Type-I neurons. The current balance equation for this model is

$$C\frac{dv_i}{dt} = -g_{Na} m^3 h(v_i - V_{Na}) - g_k n^4 (v_i - V_K) - g_l(v_i - V_l) + \epsilon g_{ij} G_{ij}(X_i, X_j) + I_i + \sigma\xi_1(t) \quad \text{(S43)}$$

where C is the membrane capacitance in $\mu F/cm^2$, vi is the membrane voltage in mV. g_{Na}, g_K and g_l are the maximum conductance per surface for the sodium, potassium and leak currents, respectively, and V_{Na}, V_K and V_l are constants which determine the corresponding reversal potentials [S20].

m, and h are the activation variable for the inactivation variable for sodium current, respectively; and n is the activation variable for potassium current. The time course of the gating variables obey

$$\begin{aligned} m = m_\infty(V) &= \frac{\alpha_m(V)}{\alpha_m(V) + \beta_m(V)} \\ \frac{dh}{dt} &= \phi\left[\alpha_h(V)(1-h) - \beta_h(V)h\right] \\ \frac{dn}{dt} &= \phi\left[\alpha_n(V)(1-n) - \beta_n(V)n\right] \end{aligned}$$

with

$$\begin{aligned}
\alpha_m(V) &= -0.1(V+35)/(exp(-0.1(V+35))) - 1) \\
\beta_m(V) &= 4exp(-(V+60)/18) \\
\alpha_h(V) &= 0.07exp(-(V+58))/20) \\
\beta_h(V) &= 1/(exp(-0.1(V+28)+1) \\
\alpha_n(V) &= -0.01(V+34)/(exp(-0.1(V+34)) - 1 \\
\beta_n(V) &= 0.125exp(-(V+44)/80).
\end{aligned} \tag{S44}$$

In the simulations we have let $\sigma = 0.005$ μA/cm^2, $g_{12} = 0$ mS/cm^2, $g_{21} = 0.5$ mS/cm^2, and $I = 1 + \varepsilon\Delta I/2$ μA/cm^2.

[S1] A. L. Hodgkin and A. F. Huxley, The Journal of physiology, **117**, 500 (1952).
[S2] L. Lapicque, J. Physiol. Pathol. Gen, **9**, 620 (1907).
[S3] E. M. Izhikevich and B. Ermentrout, Scholarpedia, **3**, 1487 (2008).
[S4] M. A. Schwemmer and T. J. Lewis, in *Phase Response Curves in Neuroscience* (Springer, 2012) pp. 3–31.
[S5] I. Malkin, Gostexizdat, Moscow (1949).
[S6] I. Malkin, Gostexizdat, Moscow, **541** (1956).
[S7] J. C. Neu, SIAM Journal on Applied Mathematics, **37**, 307 (1979).
[S8] G. B. Ermentrout and N. Kopell, SIAM journal on Mathematical Analysis, **15**, 215 (1984).
[S9] A. Winfree, Journal of Mathematical Biology, **1**, 73 (1974).
[S10] F. C. Hoppensteadt and E. M. Izhikevich, *Weakly connected neural networks*, Vol. 126 (Springer New York, 1997).
[S11] Y. Kuramoto, *Chemical oscillations, waves, and turbulence* (Courier Corporation, 2003).
[S12] Y. Kuramoto, Berlin: Springer, 1984. (1984).
[S13] Y. Kuramoto, Physica D: Nonlinear Phenomena, **50**, 15 (1991).
[S14] X.-J. Wang and G. Buzsáki, The journal of Neuroscience, **16**, 6402 (1996).
[S15] H. Risken, *The Fokker-Planck equation: methods of solution and applications* (Springer-Vlg, 1985).
[S16] R. L. Stratonovich, *Topics in the theory of random noise*, Vol. 2 (CRC Press, 1967).
[S17] N. G. Van Kampen, *Stochastic processes in physics and chemistry*, Vol. 1 (Elsevier, 1992).
[S18] B. Pfeuty, G. Mato, D. Golomb, and D. Hansel, Neural Computation, **17**, 633 (2005).
[S19] T. W. Troyer and K. D. Miller, Neural Computation, **9**, 971 (1997).
[S20] S. Sadeghi and A. Valizadeh, Journal of computational neuroscience, **36**, 55 (2014).
